# Risk of Inflammatory Bowel Disease According to Self-Rated Health, Pregnancy Course, and Pregnancy Complications: A Study within the Danish National Birth Cohort

**DOI:** 10.1371/journal.pone.0059698

**Published:** 2013-03-19

**Authors:** Maria C. Harpsøe, Kristian Tore Jørgensen, Morten Frisch, Tine Jess

**Affiliations:** 1 Department of Epidemiology Research, Statens Serum Institut, Copenhagen, Denmark; 2 Department of Occupational and Environmental Medicine, Bispebjerg Hospital, Copenhagen University Hospital, Copenhagen, Denmark; Charité-Universitätsmedizin Berlin, Germany

## Abstract

**Background:**

Poor self-rated health (SRH) has been connected to immunological changes, and pregnancy complications have been suggested in the etiology of autoimmune diseases including inflammatory bowel disease (IBD). We evaluated the impact of self-rated pre-pregnancy health and pregnancy course, hyperemesis, gestational hypertension, and preeclampsia on risk of IBD.

**Methods:**

Information was collected by questionnaires from The Danish National Birth Cohort (enrolment 1996–2002) at 16^th^ and 30^th^ week of pregnancy and 6 months postpartum. A total of 55,699 women were followed from childbirth until development of IBD (using validated National Hospital Discharge Register diagnoses), emigration, death, or end of follow-up, 31^st^ of October, 2011. Hazard ratios (HR) with 95% confidence intervals (CI) were calculated using Cox proportional hazards models adjusting for age and evaluating pre-pregnancy BMI, parity, alcohol and tobacco consumption, and socio-occupational status as potential confounders.

**Results:**

Risk of IBD increased with decreasing level of self-rated pre-pregnancy health (p = 0.002) and was elevated in women with poor self-rated pregnancy course (HR, 1.61, 95% CI 1.22–2.12). Associations persisted for more than 5 years postpartum. Hyperemesis and preeclampsia were not significantly associated with risk of IBD.

**Conclusions:**

This is the first prospective observational study to suggest that poor self-rated health – in general and in relation to pregnancy – is associated with increased risk of IBD even in the long term though results needs further confirmation. Symptoms of specific pregnancy complications were, on the other hand, not significantly associated with risk of IBD.

## Introduction

A steady increase in incidence of inflammatory bowel disease (IBD) has been seen in the northern developed countries during the past decades [1]–[3] with a peaking incidence in the reproductive years [3]. Susceptibility to IBD has been found to be influenced by dietary and life style factors [2] as well as by genetics [4], and serum antibodies have been found to predict onset of IBD several years before onset of clinical symptoms [5]. Still, the mechanisms behind development of IBD are incompletely understood.

Due to the higher incidence of IBD in reproductive years, the possible role of pregnancy in the aetiology of IBD is of interest, but most studies have investigated outcomes in patients with existing IBD [6], [7]. Self-rated pre-pregnancy health and pregnancy complications like hyperemesis, gestational hypertension, and preeclampsia might be linked with risk of later disease development. Poor self-rated health (SRH) has been connected to systemic inflammatory changes [8]–[11], and mental disturbances like chronic stress, conceivably due to its association with poor SRH, has been suggested a major risk factor in the pathogenesis of IBD [12]. However, to our knowledge, associations of SRH, in general and in relation to pregnancy, with later development of IBD have not been investigated in a prospective observational study.

In contrast, hyperemesis, a pregnancy complication characterised by nausea, vomiting, and weight loss initiated within 9 week's gestation [13], has previously been suggested to increase the risk of both Crohn's disease (CD) and ulcerative colitis (UC), whereas no such association has been observed for gestational hypertensive disorders like preeclampsia [14], despite the fact that preeclampsia, to a greater extent than hyperemesis, is followed by a cascade of systemic inflammatory responses [15] and circulatory disturbances [16], [17].

On this background, the present study aimed to explore the possible influence of *self-rated pre-pregnancy health, self-rated pregnancy course, hyperemesis, gestational hypertension*, and *preeclampsia* on risk of IBD using a large national cohort study.

## Materials and Methods

### Ethics Statement

The study was purely register-based and followed the regulations and instructions set up by the Danish Data Protection Agency (approval no. 2008-41-2374).

### Study population

The study was based on an initial sample of women in the Danish National Birth Cohort (DNBC, www.dnbc.dk) completing 86,054 full-term pregnancies (>37 weeks of gestation). Information was retrieved from the DNBC using telephone-interviews from on average the 16^th^ week (interview 1) and 30^th^ week (interview 2) of pregnancy and 6 months after delivery (interview 3) [18]. Women were enrolled in the DNBC during 1996–2002 with one or more pregnancies and participated in at least one of the three interviews. The characteristics of excluded women were similar to included women according to age, parity, smoking and alcohol consumption, and socio-occupational status. The questionnaires for the interviews were developed in consultation with external experts and were subject to several pilot tests over a four year period [18]. Additionally, a possible interviewer effect due to interviewers' health beliefs and personal habits has been investigated and found negligible [19]. Using the unique 10-digit identification number ascribed to all Danish inhabitants at birth, the DNBC participants were linked to the National Hospital Discharge Register to identify patients with IBD (International Classification of Disease 8^th^ edition [ICD-8] codes: UC, 56319, 56904; CD, 5630 and 10^th^ edition [ICD-10] codes: UC, K51; CD, K50). Women who did not answer the underlying questions to the exposure variables and women with self-reported bulimia or antihypertensive drug use before pregnancy were excluded resulting in a cohort of 56,108 women. Among these women, we excluded 399 women with a first IBD diagnosis recorded in the Danish National Patient Register before the date of birth of the child and 10 women with self-reported IBD at interview 1. Consequently, the cohort included 55,699 women.

### Exposure variables

Exposure variables were defined as *self-rated pre-pregnancy health* (“very good” [reference], “fair”, “poor” [covering “not so good”]) using interview 1, “How would you characterise your health in general?”, *self-rated pregnancy course* (“well or fair” [covering “very well”, “well” and “fair”, reference], and “poor or very poor”) using interview 2 and 3, *pregnancy-associated hypertension* (normal blood pressure [reference], gestational hypertension, preeclampsia) using interview 2 and 3, and *hyperemesis* (“no nausea, vomiting, or weight loss” [reference], “nausea”, “vomiting without weight loss”, “vomiting and weight loss”) using interview 2. *Self-rated pregnancy course* was categorized as “well or fair” when the answers showed no indication of a “poor” pregnancy course in any of the two interviews but the women had to have answered the question in interview 3 taking place six months after delivery. Indications of a “poor” pregnancy course in any of the two interviews led to categorization as “poor or very poor” *self-rated pregnancy course*.

### Potential confounders

The analyses were all adjusted for the woman's age at interview 1 (a priori confounder; <20, 20–24, 25–29 [reference], 30–34, 35–39, and 40+ years) and further evaluated for other potential confounders listed in Table 1, including parity, smoking during and after pregnancy reported in interview 1, 2, or 3 (as an expression of smoking before exposure since few are assumed to start smoking during pregnancy), alcohol consumption prior to pregnancy, pre-pregnancy body mass index (BMI, kg/m^2^), and socio-occupational status. BMI was examined as a potential confounder since obesity is considered a condition of chronic low-grade systemic inflammation [20], [21]. Potential confounders were retained in the analysis only if hazard ratios changed more than 10% which was the case only for one variable, namely socio-occupational status in the analysis of *self-rated pre-pregnancy health* and risk of Crohn's disease.

**Table 1:** Cohort characteristics (n = 55,699).

**Maternal age** *(mean yrs*±*SD)*	30.0±4.3
**Parity** *(n [%])*	
Primipara	27,816 (49.9)
1 child	18,893 (33.9)
2+ children	8,958 (16.1)
Missing	32 (0.1)
**BMI** *(kg/m^2^ [%])*	
<18.5	2,325 (4.2)
18.5–<25	37,090 (66.6)
25–<30	10,760 (19.3)
≥30	4,610 (8.3)
Missing	914 (1.6)
**Alcohol consumption per week prior to pregnancy** *(n [%])*	
0 units	6,877 (12.4)
1–7 units	43,259 (77.7)
8+ units	5,307 (9.5)
Missing	256 (0.5)
**Smoking during and after pregnancy reported in interview 1, 2, or 3** *(n [%])*	15,113 (27.1)
**Socio-occupational status** *(n [%])*	
Long edu/leaders in large companies	19,147 (34.4)
Middle long edu/leaders in small companies	17,907 (32.2)
Short edu/vocational/under edu	16,455 (29.5)
Unskilled/other work/unemployment benefits	1,773 (3.2)
On state welfare	230 (0.4)
Missing	187 (0.3)
***Exposure variables*** * (n [%])*	
Self-rated pre-pregnancy health – poor	1,847 (3.3)
Self-rated pregnancy course – poor or very poor	6,933 (12.5)
Hyperemesis	5,295 (9.5)
Gestational hypertension	5,750 (10.3)
Preeclampsia	1,991 (3.6)
***Outcome variables*** * (n [%])*	
Ulcerative colitis	251 (0.45)
Crohn's disease	89 (0.16)
Inflammatory bowel disease overall	340 (0.61)

### Statistical analyses

The cohort was followed from the date of delivery, or in cases of stillbirth at date of interview 1 adding 24 weeks, and until development of IBD (1^st^ hospital contact), emigration, death, or end of follow-up on 31^st^ of October, 2011. Hazards ratios (HR) of first hospitalization for IBD with 95% confidence intervals (CI) were calculated using Cox proportional hazards models. In sensitivity analyses it was tested whether mutual adjustment for *self-rated pre-pregnancy health* and *pregnancy course* changed the estimates. Additional analyses with stratification on time of follow up (less or more than five years after birth) were generated for *self-rated pre-pregnancy health* and *self-rated pregnancy course* with tests of homogeneity within the same period and between the two time periods. A p-value <0.05 was considered statistically significant. All statistical tests were performed using SAS software version 9.2 (SAS Institute Inc., Cary, NC, USA).

## Results

The women in the cohort were followed for a mean time of 11.1±1.6 years since delivery. Among the 55,699 women of whom 0.6% developed IBD (n = 340) the mean time from delivery until diagnosis was 4.7±3.1 years for IBD, 4.4±3.2 years for UC, and 5.2±3.0 years for CD. Altogether, 3.3% of women reported “poor” *self-rated pre-pregnancy health*, 12.5% of the women reported “poor or very poor” s*elf-rated pregnancy course*, and 9.5% reported *hyperemesis* (vomiting and weight loss), 10.3% *gestational hypertension*, and 3.6% *preeclampsia* (Table 1).

A decreasing level of *self-rated pre-pregnancy health* (p = 0.002) and “poor or very poor” *self-rated pregnancy course* (HR, 1.61; 95% CI, 1.22–2.12) were significantly associated with later development of IBD (Table 2). These observations were primarily explained by a greater risk of UC in individuals with decreasing level of *self-rated pre-pregnancy health* (p = 0.02), and “poor or very poor” *self-rated pregnancy course* (HR, 1.68; 95% CI, 1.22–2.30) (Table 3).

**Table 2:** Hazard ratios (HR) of overall inflammatory bowel disease according to self-rated pre-pregnancy health, self-rated pregnancy course, and pregnancy complications among 55,699 women in the Danish National Birth Cohort.

	Inflammatory bowel disease		
	No	Yes	HR^§^ (95% CI)
**Self-rated pre-pregnancy health**			
Very good	29,291	146	1 (reference)
Fair	24,235	180	1.47 (1.18–1.83)
Poor	1,833	14	1.53 (0.88–2.65)
*Test for homogeneity*			*p = 0.002*
**Self-rated pregnancy course**			
Well or fair	48,489	277	1 (reference)
Poor or very poor	6,870	63	1.61 (1.22–2.12)
*Test for homogeneity*			*p<0.001*
**Hyperemesis during pregnancy**			
No nausea, vomiting, or weight loss	21,891	123	1 (reference)
Nausea	19,390	120	1.11 (0.87–1.43)
Vomiting	8,820	60	1.21 (0.89–1.64)
Vomiting and weight loss	5,258	37	1.23 (0.85–1.77)
*Test for homogeneity*			*p = 0.56*
**Pregnancy-associated hypertension**			
Normal blood pressure	47,671	287	1 (reference)
Hypertension	5,713	37	1.07 (0.76–1.50)
Preeclampsia	1,975	16	1.31 (0.79–2.18)
*Test for homogeneity*			*p = 0.55*

CI, confidence interval; ^§^ adjusted for age.

**Table 3:** Hazard ratios (HR) of ulcerative colitis (UC) and Crohn's disease (CD) according to self-rated pre-pregnancy health, self-rated pregnancy course, and pregnancy complications among 55,699 women in the Danish National Birth Cohort.

	Ulcerative colitis			Crohn's disease		
	No	Yes	HR^a^ (95% CI)	No	Yes	HR^a^ (95% CI)
**Self-rated pre-pregnancy health**						
Very good	29,327	110	1 (reference)	29,401	36	1 (reference)
Fair	24,285	130	1.42 (1.10–1.83)	24,365	50	1.56^b^ (1.02–2.41)
Poor	1,836	11	1.60 (0.86–2.98)	1,844	3	1.12^b^ (0.34–3.64)
*Test for homogeneity*			*p = 0.02*			*p = 0.12*
**Self-rated pregnancy course**						
Well or fair	48,563	203	1 (reference)	48,692	74	1 (reference)
Poor or very poor	6,885	48	1.68 (1.22–2.30)	6,918	15	1.43 (0.82–2.49)
*Test for homogeneity*			*p<0.001*			*p = 0.21*
**Hyperemesis during pregnancy**						
No nausea, vomiting or weight loss	21,920	94	1 (reference)	21,985	29	1 (reference)
Nausea	19,425	85	1.02 (0.76–1.37)	19,475	35	1.41 (0.86–2.31)
Vomiting	8,836	44	1.16 (0.81–1.65)	8,864	16	1.37 (0.74–2.52)
Vomiting and weight loss	5,267	28	1.22 (0.80–1.86)	5,286	9	1.24 (0.59–2.63)
*Test for homogeneity*			*p = 0.73*			*p = 0.56*
**Pregnancy-associated hypertension**						
Normal blood pressure	47,747	211	1 (reference)	47,882	76	1 (reference)
Hypertension	5,722	28	1.11 (0.75–1.65)	5,741	9	0.96 (0.48–1.91)
Preeclampsia	1,979	12	1.36 (0.76–2.43)	1,987	4	1.19 (0.44–3.27)
*Test for homogeneity*			*p = 0.54*			*p = 0.93*

CI, confidence interval; ^a^adjusted for age; ^b^additionally adjusted for socio-occupational status.

The associations between symptoms of *hyperemesis, gestational hypertension*, *preeclampsia* and development of IBD did not reach statistical significance (Table 2 and 3).

In sensitivity analyses, mutual adjustment for self-rated pre-pregnancy health and pregnancy course did not change the estimates markedly. Stratification on risk of IBD according to time since pregnancy, women with “poor” *self-rated pre-pregnancy health* had an increased risk of both UC and CD after five years, with a combined estimate for IBD of 2.29 (95% CI, 1.14–4.60; Table 4). The risk of IBD overall and UC in particular remained significantly increased after 5 years in women with a “poor or very poor” *self-rated pregnancy course*. Generally, associations of poorly rated *pre-pregnancy health* and *pregnancy course* with IBD risk were stronger in the period ≥5 years than the period <5 years after delivery (Table 4).

**Table 4:** Hazard ratios with 95% confidence interval of inflammatory bowel disease, ulcerative colitis, and Crohn's disease according to self-rated pre-pregnancy health and self-rated pregnancy course before and after 5 years of follow up among 55,699 women in the Danish National Birth Cohort.

	Inflammatory bowel disease^a^ (n = 340)				Ulcerative colitis^a^		Crohn's disease^a^	
	N^c^	<5 years	N^c^	5+ years	<5 years	5+ years	<5 years	5+ years
**SRH**								
Very good	83	1 (reference)	63	1 (reference)	1 (reference)	1 (reference)	1 (reference)	1 (reference)
Fair	97	1.40 (1.04–1.87)	83	1.58 (1.13–2.19)	1.45 (1.04–2.03)	1.38 (0.93–2.03)	1.18 (0.65–2.14)	2.10^b^ (1.12–3.93)
Poor	5	0.96 (0.39–2.36)	9	2.29 (1.14–4.60)	1.29 (0.52–3.21)	2.02 (0.86–4.71)	No events	2.67^b^ (0.77–9.26)
*Homogeneity test*		*p = 0.07*		*p = 0.01*	*p = 0.09*	*p = 0.12*	*p = 0.87*	*p = 0.049*
**SR pregnancy**								
Well or fair	152	1 (reference)	125	1 (reference)	1 (reference)	1 (reference)	1 (reference)	1 (reference)
Poor or very poor	33	1.54 (1.06–2.24)	30	1.70 (1.14–2.53)	1.59 (1.04–2.43)	1.79 (1.12–2.86)	1.37 (0.61–3.08)	1.48 (0.69–3.18)
*Homogeneity test*		*p = 0.03*		*p = 0.01*	*p = 0.03*	*p = 0.01*	*p = 0.45*	*p = 0.31*

a Adjusted for age; ^b^additionally adjusted for socio-occupational status; ^c^number of IBD cases.

Test for homogeneity between periods were insignificant for all outcomes.

SRH: Self-rated pre-pregnancy health; SR pregnancy; Self-rated pregnancy course.

## Discussion

This study of 55,699 Danish women followed prospectively for up to a decade after pregnancy suggests that decreasing level of *self-rated pre-pregnancy health* and poor *self-rated pregnancy course* are linked to increased risk of IBD even more than 5 years after ended pregnancy. This increased risk even seems to be greater in the long-term than within the first 5 years following pregnancy. Mutual adjustment for *self-rated pre-pregnancy health* and *pregnancy* course did not change the estimates. On the other hand, specific pregnancy complications such as *hyperemesis, gestational hypertension*, and *preeclampsia* were not significantly associated with risk of IBD.

### Strengths and limitations

A major strength of this study was that our data stem from a large, national birth cohort of women followed prospectively after pregnancy combined with high-quality data from the National Hospital Discharge Register, in which diagnoses of CD and UC have been found highly complete (94%) with validity estimates for registered CD and UC diagnoses of 97% and 90%, respectively, using a pathology register as reference [22]. Further, unpublished data from a Danish inception cohort study found only 8.6% of UC patients and 1.0% of patients with CD to be diagnosed outside Danish hospitals [3]. Accordingly, our findings are most likely applicable to both mild and severe cases of IBD. Another asset is the novelty of our study. Only one previous study has addressed pregnancy complications (but not SRH) and subsequent risk of IBD [14], but that study was hospital-based and limited by the fact that not all pregnancy complications lead to hospital referral. An additional strength of our study is that we had access to information on *self-rated pre-pregnancy health* and *pregnancy course* collected *prior to* diagnosis of IBD, hence avoiding recall bias.

The study also has limitations to consider. Exposure variables were based on self-reports which may have led to misclassifications of pregnancy complications and should be kept in mind when interpreting results. On the other hand, the milder cases of *hyperemesis* and *gestational hypertension* are most likely not hospitalised why self-reporting is assumed to identify more cases and hence a more comprehensive spectrum of our exposures than pure register information. Further, a validation study of self-reported preeclampsia in the DNBC (compared to information in the Danish National Hospital Discharge Register and manually searched hospital charts) showed a sensitivity of 72.6% and specificity of 98.6% of self-reported preeclampsia in this cohort. When adding a question concerning “medication due to hypertension in pregnancy” to identify further preeclampsia cases, the sensitivity decreased to 27.4% [23]. We therefore chose not to include this question. Another limitation is the definition of hyperemesis as vomiting and weight loss, which did not necessarily occur within the same time period during pregnancy. However, since weight loss is uncommon during pregnancy, these symptoms were assumed to go hand in hand. With respect to self-rated pregnancy course, this variable was as an extension of SRH reflecting a specific and vulnerable period in the woman's life, i.e. pregnancy. Nevertheless, interpretations of both self-rated pregnancy course and SRH should be made with caution due to its subjective nature. However, SRH has been utilized in numerous studies and found to be strongly correlated with both mortality [24]–[28] and morbidity [25], [29]. Validation of SRH specifically within DNBC has not been performed, but the specific SRH-question has been subject to the above mentioned pilot tests and has been used in a previous study [30]. Further, the phrasing of the SRH-question in the DNBC is similar to phrasings used in numerous other studies and forms part of one of the most widely used instruments in measurement of health status, the Short Form (36) Health Survey [25]. The interview question used for SRH was performed as a 3 opposite to a 5 or 7 point scale which is more often used, but answers to the various SRH questions applied in 5 and 7 point scales have been found strongly correlated in a study by Eriksson and colleagues [31]. The same study observed that especially younger women between 18–44 years of age often rate their health slightly poorer than men supporting the reasoning in choosing cohort participants of the same sex and age range.

### Self-rated pre-pregnancy health and pregnancy course

To our knowledge, this is the first prospective observational study to investigate the effect of self-rated pregnancy course and SRH on the risk of IBD [32], [33]. However, many studies have examined the effect of SRH/health-related quality of life on existing IBD in all ages [33]–[35], and previous literature further suggests a connection between mood disorders like depression and anxiety and IBD [36]. The present study observed a long-term effect (more than 5 years after pregnancy) of both *self-rated pre-pregnancy health* and *pregnancy course* on development of IBD. We consider reverse causality, in which symptoms of yet undiagnosed IBD leads to poor *self-rated health* and poor *pregnancy course*, unlikely, due to the long-term effect of poor SRH on risk of IBD and because the median time from symptom onset to IBD diagnosis is less than a year in Denmark [1]. Nonetheless, subclinical symptoms leading to a poorly rated health should be considered. Further, the long-term effect of both “fair” and “poor” SRH compared to “very good” SRH on risk of IBD shown after 5 years was found significant only for “fair” SRH in the first 5 years. This finding could be due to lack of power considering a low number of cases (n = 5) with poor SRH in the first 5 years but the fact that people not necessarily apply the same frame of reference [25] could be part of the explanation as well. The choice of a dichotomous categorization of *self-rated pregnancy course* was due to an interest in “poor” pregnancy course rather than in the different degrees of a “fair” or “well” *self-rated pregnancy course*.

Previous studies have shown an association between poor SRH and increased serum levels of inflammatory cytokines [8], [9], [11], [26], but mainly tested on patients with already diagnosed conditions like asthma, allergy, cardiovascular disease, and other diseases likely to affect the level of cytokines in serum. The generalizability of these studies may thereby be limited but a recent study examining employees *without* a definitive health condition confirmed an association between poor SRH and inflammatory response [10]. Further, SRH has been found to be a more robust predictor of cytokine levels than physical health, especially in women [9], and inflammatory markers like interleukin-6 has been found to increase with age [11]. This underlines the importance of studying a cohort within a narrow age range; in our study represented by women of 30.0±SD 4.3 years of age. In autoimmune diseases like RA, type 1 diabetes, and systemic lupus erythematosus, the first immunological signs are detectable up to decades before clinical diagnosis [37]–[39], and such immunological changes may, in turn, result in poor SRH as mentioned above. Whether this applies to IBD as well is unknown but the hypothesis is supported by the presence of serological markers, such as anti-neutrophil cytoplasmic antibodies and anti-*Saccharomyces cerevisiae* mannan antibodies and antibodies against *Escherichia coli* outer membrane porin C, in otherwise healthy individuals several years before diagnosis of IBD [5]. Reviews further suggest that stress can induce IBD onset through dysregulation of the brain-gut-axis (the communication between the enteric nervous system and the central nervous system), release of pro-inflammatory mediators, and changes in the gut environment [12], [33]. In accordance with our findings, a review has found stress to associate with development of CD [40] and further, there is increasing evidence that psychological stress can exacerbate the activity of already existing IBD [32], [41]. Hence, findings in our and previous studies suggest that some factor associated with poor SRH may be involved in development of IBD, either through a stress induced inflammatory pathway leading to IBD or simply because early immunological changes in IBD lead to poor SRH.

### Hyperemesis, gestational hypertension, and preeclampsia

In contrast to the recent study by Jørgensen and colleagues [14], we did not observe a significantly increased risk of IBD in women with symptoms of *hyperemesis*. However, as mentioned, the previous study was based solely on register information, hence applying to hospitalised and thereby more severe cases, whereas our study used self-reported data covering both mild and severe pregnancy complications. Though insignificant, we cannot exclude a trend towards higher risk of IBD with increasing symptom severity of *hyperemesis* and negative findings in our study may relate to lack of power. We can therefore not exclude that severe *hyperemesis* (i.e. the subset present in hospital series) is associated with development of IBD. Concerning preeclampsia, our results are in accordance with those of Jørgensen and colleagues showing no association with IBD, despite known immunological and circulatory alterations in women with preeclampsia [15]–[17].

In conclusion, our large and prospective national birth cohort study found decreasing level of *self-rated pre-pregnancy health* and poor *pregnancy course* to be independently associated with increased risk of IBD more than 5 years following pregnancy, whereas the study did not confirm that symptoms of *hyperemesis* or pregnancy-related hypertensive disorders like *preeclampsia* are associated with later development of IBD. Mechanisms behind the suggested association between poor SRH and IBD are unknown but evidence of an association between poor SRH and immunological changes is growing. The relationship between SRH – both pregnancy-related and in general – and subsequent risk of IBD needs cautious evaluation and further investigation.
